# Role of Complement Anaphylatoxins (C3a and C5a) in Lymphoma: A Systematic Review

**DOI:** 10.7759/cureus.91213

**Published:** 2025-08-28

**Authors:** Konstantinos Skarentzos, Pinelopi Vryttia, Sotiris Papageorgiou, Periklis Foukas

**Affiliations:** 1 Second Department of Pathology, National and Kapodistrian University of Athens, Medical School, "Attikon" University Hospital, Athens, GRC; 2 Second Department of Internal Medicine and Research Institute, National and Kapodistrian University of Athens, Medical School, "Attikon" University Hospital, Athens, GRC

**Keywords:** anaphylatoxins, c3a, c5a, complement, lymphoma

## Abstract

Tumor cell survival, growth, and proliferation are related to the interactions between tumor cells and immune cells. Anaphylatoxins C3a and C5a seem to be involved in these cell-to-cell interactions. The aim of this study is to investigate whether the increase of C3a and/or C5a levels induces the proliferation of lymphoma cells.

The population consisted of naïve lymphoma patients or human lymphoma cell lines. Comparisons of C3a and C5a levels between lymphoma patients and a healthy control group and between lymphoma cell lines and control media were acceptable. Studies that demonstrated outcomes regarding patients' survival and the growth and proliferation of lymphoma cells were included. A search was conducted on MEDLINE (through PubMed), Cochrane, and Scopus on January 2, 2025. Two independent reviewers performed the quality assessment using the Oral Health Assessment Tool (OHAT) modified for in vitro studies. Results were descriptively presented.

Through a search of the aforementioned libraries, 852 studies were identified. Four studies were eligible: two studies used only cell lines, one used a cell line and paraffin blocks from 58 patients, and the other used serum from 39 naïve B-cell lymphoma patients. Regarding in vitro studies, it was shown that C5a induced neutrophil-mediated antibody-dependent tumor cell lysis. Elevated levels of C5a in co-culture with M2 macrophages lead to increased lymphoma cell proliferation, and patients with high densities of M2 macrophages in the tumor microenvironment (TME) had poor prognosis.

Regarding the limitations of this review, only a few articles were found to study this hypothesis. Evidence of a correlation between anaphylatoxin levels and lymphoma cell proliferation raises the need for further investigation in order to understand the role of complement in B-cell lymphoma development and progression. In summary, anaphylatoxins, especially C5a, appear to play a role in the activation of lymphoma cells both as mediators in cell-cell interaction and as auxiliary molecules for the lysis of these cells via antibody-dependent cellular cytotoxicity.

## Introduction and background

The tumor microenvironment (TME) plays a determinative role in tumor survival and progression through a constant interaction between cancer cells and proximal immune cells. Regarding innate immunity, macrophages can be polarized into M1 or M2 functional states, with the latter causing immunosuppression through the secretion of cytokines (like IL-4) in the TME. Similarly, N2 neutrophils promote tumor growth. Depending on the signals from the TME, tumor-infiltrating dendritic cells (TIDCs) could stimulate Th2, Th17, and T regulatory responses, enhancing tumor suppression [1].

Anaphylatoxins are small protein fragments formed by a series of enzymatic reactions that take place during the complement cascade: the activation of classical, lectin, or alternative pathway causes the formation of C3a and C5a. Specifically, C3 convertases (C4b2b) cleave C3 in order to generate the anaphylatoxin C3a and the opsonin C3b. C5 convertases (C4b2b3b and C3bBb3b) are formed by the excess of C3b and cleave C5 to generate C5a and C5b. C5a is a strong chemoattractant and plays a significant role in the recruitment of inflammatory cells, in the activation of phagocytic cells and release of granule-based enzymes, and in the generation of oxidants, all of which may lead to innate immune functions or tissue damage [2]. Peng et al. have concluded that both C3a and C5a anaphylatoxins act as cell activators and contribute to the pathogenesis of several inflammatory and autoimmune diseases such as sepsis, systemic lupus erythematosus, antiphospholipid syndrome, ischemia/reperfusion (I/R) injury, and asthma [3]. However, the role of C3a and C5a in the TME of lymphoma has not been thoroughly studied yet.

Previous studies have demonstrated several interconnections between the complement system and cancer. Activation of the complement system in the TME is proven to enhance tumor growth via several immune and non-immune functions in both plasma and extravascular interstitial tissue [4]. It has been shown that anaphylatoxins C3a and C5a may promote tumor growth through the downregulation of antitumor T-cell responses. In addition, there are many ongoing phase I/II clinical trials about novel drugs, which target C3a and C5a and their receptors [5].

Lymphomas, the malignant neoplasms of lymphocytes, are classified broadly as Hodgkin and non-Hodgkin, with more than 100 entities in the last World Health Organization (WHO) classification. Because of the heterogeneity of this malignancy, lymphoma patients may present with various symptoms. Nevertheless, a "typical" lymphoma patient usually presents with painless lymphadenopathy, fever, weight loss, and night sweats. Lymphomas with a high proliferation index are associated with poor prognosis. Usually, this malignancy is diagnosed by a lymph node biopsy, but lymphoma could also affect the bone marrow or extranodal tissues [6,7]. Regardless of the fact that the role of complement in the effectiveness of immunotherapeutic approaches like anti-CD20 (rituximab) has been highlighted [8], only a few articles in the literature focus on the effect that anaphylatoxins have on lymphoma cells before treatment. The aim of this systematic review is to investigate whether the increase of C3a and/or C5a levels induces the proliferation of lymphoma cells.

## Review

Methods

Eligibility Criteria

The protocol for this systematic review received approval from the International Prospective Register of Systematic Reviews (PROSPERO) (registration number: CRD420250654200). The inclusion criteria were based on the PICO (Population, Intervention, Comparison, and Outcome) model. The population consisted of lymphoma patients or human lymphoma cell lines. Since our aim was to study the pathophysiology of the disease, every intervention that could interfere with C3a or C5a was excluded (e.g., administration of immunotherapy). Comparisons of C3a and C5a levels between lymphoma patients and a healthy control group and between lymphoma cell lines and control media were acceptable. Studies that demonstrated outcomes regarding patients' survival and the growth and proliferation of lymphoma cells were included. Articles written only in English were accepted.

On the other hand, studies about animal models and genomes were excluded. Studies that provide data about C3a and C5a levels in relation to drug reactions in lymphoma patients were excluded. Moreover, conference abstracts and other non-peer-reviewed studies were excluded. Editorials, short communications, case report studies, and case series were excluded. For obvious reasons, other systematic reviews and any other kind of review were excluded.

Search Strategy, Selection Process, and Data Extraction

A thorough search was conducted on MEDLINE (through PubMed), Cochrane, and Scopus. We searched articles from inception until January 2, 2025. The following search strategy was used: "(C3a OR C3aR OR C5a OR C5aR OR Anaphylatoxin OR complement peptide) AND lymphoma". Two reviewers, blinded to each other's decisions, performed title and abstract screening according to the aforementioned eligibility criteria. Any conflict was resolved by discussion. Then, full-text screening was independently conducted by the same two reviewers. Reasons for exclusion were reported, and consensus was reached via conversation.

Data extraction was performed by K.S. and P.V., again blinded to each other's decisions. Data were collected about authors' names and affiliations, year of publication, population demographics, human lymphoma cell lines' characteristics, experimental procedures (e.g., co-culture with lymphoma cell lines and macrophages), survival rates of lymphoma patients, C3a and C5a levels, and lymphoma cells' growth, proliferation, apoptosis, and nuclear size. Since the extracted data did not qualify for meta-analysis, a narrative synthesis was applied.

Risk of Bias

Two reviewers (K.S. and P.V.) independently performed the quality assessment using the Oral Health Assessment Tool (OHAT) [9] modified for in vitro studies [10]. This quality assessment tool consisted of nine questions. Every question can be answered on a scale from "definitely low" to "definitely high".

EndNote™ 20 (Clarivate, London, United Kingdom) was used as the citation manager [11].

Results

Study Selection

**Figure 1:**
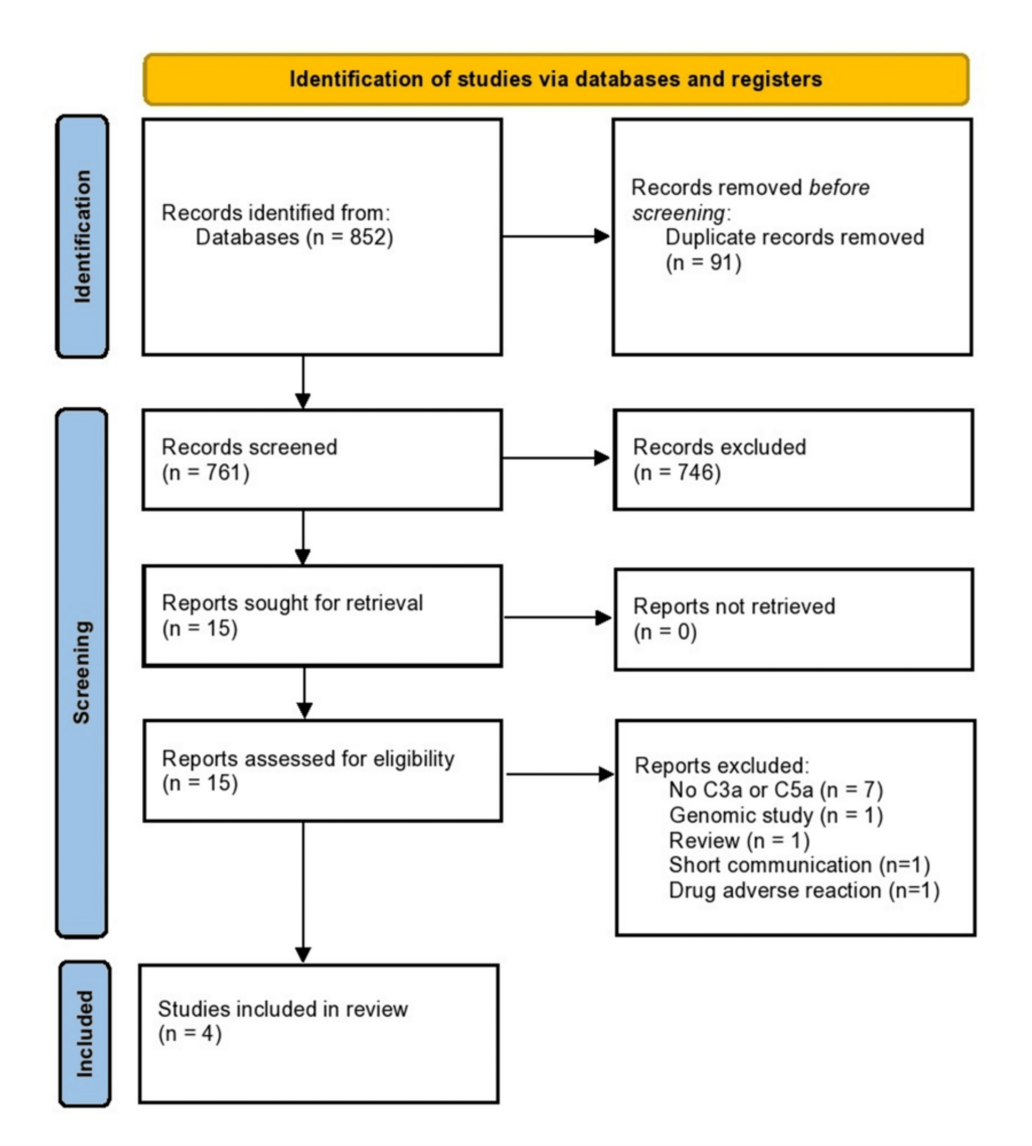
PRISMA flowchart PRISMA: Preferred Reporting Items for Systematic Reviews and Meta-Analyses

From the initial application of the aforementioned search algorithm, 852 studies were identified, of which 91 were duplicates. After duplicate removal, 761 articles remained. The articles that met our inclusion criteria in title and abstract screening were 15. At last, four studies were included in this systematic review after the process of full-text screening, with specific reasons for every excluded article (Figure 1). Most of the articles (seven) were excluded because they did not present data about C3a or C5a levels. One study was analyzing the gene of C5 but not the levels of C3a or C5a expression. Two studies were excluded as their design was not acceptable (review and short communication). One study was excluded because it analyzed the C3a levels after rituximab administration and not in relevance with lymphoma status. "Snowballing" was performed, but no new articles were included.

Study Characteristics and Results of Individual Studies

Four studies were included in this systematic review. The studies' characteristics are mentioned in Table 1. Two studies used only cell lines [12,13], one used a cell line and paraffin blocks from patients [14], and the other study used serum from lymphoma patients and compared it with a healthy control group [15].

**Table 1:** Characteristics and results of the individual studies Stat3: signal transducer and activator of transcription 3; BrdU: macrophage marker; Lym-1: a murine IgG2a monoclonal antibody that recognizes B-cell lymphoma cells; ATLL: adult T-cell leukemia/lymphoma; N/A: not applicable; PMN: polymorphonuclear neutrophil

Author (year)	Population	Cell lines	Cells studied	Anaphylatoxins and other molecules studied	Methods	Main outcomes
Bai et al. (2013) [12]	Cell lines (in vitro)	SLVL, Raji, Daudi	Lymphoma cells, M1, M2	C5a Stat3	Lymphoma cell lines (SLVL, Raji, and Daudi) were cultured alone or co-cultured with immature, M1, or M2 macrophages. The number of macrophages (using BrdU), lymphoma cells' nuclei size and proliferation, and Stat3 and C5a levels were studied	(1) Co-culture with M2 macrophages significantly induced BrdU incorporation
						(2) Co-culture with M2 macrophages significantly increased lymphoma cells' nuclei size
						(3) Lymphoma cell proliferation was elevated in co-culture with immature, M1 or M2 macrophages
						(4) Elevated C5a levels in co-culture with M2 macrophages activated Stat3 and increased lymphoma cell proliferation
						(5) Stat3 downregulation suppressed lymphoma cell activation by C5a
Ottonello et al. (1996) [13]	Cell lines (in vitro)	Raji	Lymphoma cells	C5a TNF-α, IGF-1, IL-8, γ-IFN, IL-2	Lymphoma cell line (Raji) was cultured with mononuclear cells and neutrophils (from healthy donor's blood), with and without Lym-1 MoAb and cytokines/chemotaxins	(1) C5-deficient human serum did not promote Lym-1 antibody-dependent lysis
						(2) C5a as a mediator induces PMN-mediated Lym-1 antibody-dependent cytolysis
						(3) When C5a was added to PMNs and lymphoma cells in the absence of Lym-1 or mixed with Raji cells plus Lym-1, C5a had no effect
Komohara et al. (2013) [14]	Cell lines (in vitro) and paraffin blocks from 58 ATLL patients	ATLL	ATLL cells, M1, M2	C5a	Immunohistochemistry with CD68 and CD163 was used to identify M1 and M2 macrophages in paraffin-embedded tumor tissue accordingly. ATLL cell lines were cultured with macrophages	(1) Patients with higher proportion of M2 macrophages had poor prognosis
						(2) ATLL cells were significantly activated when they were co-cultured with M2 macrophages
						(3) C5a, TNF-α, GRO-a, I-309, and IL-6 significantly promoted the proliferation of lymphoma cell lines when co-cultured with M2 macrophages
Miguet et al. (2006) [15]	Serum from 39 chronic B-cell lymphoma patients (before treatment) and 20 healthy individuals (control group)	N/A	N/A	C3a	Sera test for various proteins including C3a and C4a	C3a and C4a were increased in lymphoma patients versus the control group

Bai et al.'s experiments involved the SLVL, Raji, and Daudi cell lines, which are widely used cell lines derived from splenic B-cell lymphoma with circulating villous lymphocytes (SLVL) and Burkitt lymphoma (Raji and Daudi) patients, respectively. These cell lines were cultured alone or co-cultured with immature, M1, or M2 macrophages. Then the number of macrophages (using BrdU as a macrophage marker), lymphoma cells' nuclei size and proliferation, and Stat3 and C5a levels were studied. According to this study, co-culture with M2 macrophages significantly induced BrdU incorporation. The longest length of SLVL cell nuclei, when co-cultured with M2 macrophages, was 18.8±2.7 mm. Without co-culture, the longest length of SLVL cell nuclei was 15.7±2.8 mm, proving that lymphoma cells' nuclei size was significantly increased in co-culture with M2 macrophages (p=0.003; n=20). These outcomes suggest that M2 macrophages promote lymphoma cell nuclei enlargement. Moreover, lymphoma cell proliferation was elevated in co-culture with immature, M1, or M2 macrophages. In addition, elevated levels of C5a in co-culture with M2 macrophages lead to increased lymphoma cell proliferation via Stat3 activation. The research team achieved downregulation of Stat3 protein in SLVL cultured cells by siRNA. Lymphoma cell activation by C5a was suppressed when Stat3 was downregulated [12].

Another experimental study used Raji cell line co-cultured with mononuclear cells and neutrophils from the blood of a healthy donor, Lym-1 MoAb (a murine IgG2a monoclonal antibody that recognizes B-cell lymphoma cells), and cytokines/chemotaxins. It was shown that Raji cells' lysis occurred in the presence of Lym-1 and human serum. When C5 was removed from the human serum, Lym-1 antibody-dependent lysis was not promoted. C5a induced neutrophil-mediated Lym-1 antibody-dependent lysis. When C5a was put in neutrophils and lymphoma cells without Lym-1 or mixed with Raji cells and Lym-1, C5a had no effect. Thus, C5a caused lymphoma cell lysis only when neutrophils were present, suggesting a potential role of C5a in neutrophil activation. According to the authors, neutrophil-mediated Lym-1 antibody-dependent cytolysis was 5.3±8.0 (n=17) with 2.6 (1.1-9.4) in the absence of C5a and 11.6±9.0 (n=17) with 11.4 (7.0-16.3) in the presence of C5a. Cytolysis in the absence of C5a versus in the presence of C5a was statistically significant (p=0.0103) [13].

The last experimental study involved adult T-cell leukemia/lymphoma (ATLL) cell lines and paraffin-embedded tumor samples from 58 ATLL patients. The median age was 65 years, and 34 out of 58 patients were males. CD68 was used as an M1 macrophage marker, while CD163 was used as an M2 macrophage marker. Two pathologists independently evaluated the infiltration of M1 and M2 macrophages. ATLL cell lines were cultured with macrophages. Patients were divided into M2 low and M2 high groups, and the cutoff was set at 75% of CD163-positive cells of tumor-associated macrophages. This study showed that patients with higher densities of M2 macrophages had a poor prognosis (p=0.046). Proliferation of ATLL cells was significantly promoted when they were co-cultured with M2 macrophages. Last but not least, the involvement of soluble factors like C5a in the interaction between lymphoma cells and M2 macrophages was studied. It turned out that C5a, TNF-α, growth-related oncogene (GRO)-a, CCL1/I-309, and interleukin-6 significantly promoted the proliferation of lymphoma cell lines when co-cultured with M2 macrophages [14].

The study of Miguet et al. used serum from 39 naïve chronic B-cell lymphoma patients and 20 healthy individuals. Surface-enhanced laser desorption ionization (SELDI) mass spectrometer ProteinChip technology was applied in order to identify different proteins in the serum. This methodology is applied, aiming to discover new biomarkers. The research team identified all peptides and proteins that were significantly different between patients and healthy individuals. In order to avoid biases arising from therapy (immunotherapy and/or chemotherapy), serum and body fluid were obtained at the moment of diagnosis. Several serum proteins were studied, including anaphylatoxins C3a and C4a. It was shown that C3a and C4a were present with intense peaks in lymphoma patients but not in the control group. Specifically, these two peaks were 8936 Da and 8611 Da (1 Da=1 g/mol). Since C3a protein is 8938 Da and C4a is 8608 Da, they both fit very well with the experimental SELDI mass at 8936 Da and 8611 Da, respectively. The authors suggested that protein fragments or modified forms of common serum proteins could be used as potential diagnostic markers. C3a and C4a could possibly be useful for the diagnosis of B-cell lymphoma, since they were not observed in the serum of healthy individuals, but they were identified in the serum of naïve chronic B-cell lymphoma patients with major peaks [15]. 

Risk of Bias

The results of the risk of bias are shown in Table 2. All of the studies were characterized by a definitely low risk of bias in most questions. Regarding questions 1 and 2, "Was the administered dose or exposure level adequately randomized?" and "Was allocation to study groups adequately concealed?", in these experimental studies [12-14], there is no need for randomization or concealment because they consist of homogeneous cell lines. For the same questions, Miguet et al. [15] received a probably low risk of bias score, because serum samples were collected at the time of diagnosis, before any treatment, and it was not possible to be concealed. Experimental conditions are described thoroughly in all studies, and they seem to be identical. A laboratory equipment with an automated system was used in these studies [12,13,15], thus eliminating the need for personnel to be blinded to the study group. In Komohara et al., a laboratory system was also used, and personnel were blinded when evaluating immunohistochemistry [14]. Lastly, questions 5-9 were adequately answered, so the aforementioned studies were characterized by a definitely low risk of bias. 

**Table 2:** Quality assessment ++: definitely low risk of bias; +: probably low risk of bias; -: probably high risk of bias or not reported; --: definitely high risk of bias

Author (year)	1. Was the administered dose or exposure level adequately randomized?	2. Was allocation to study groups adequately concealed?	3. Were experimental conditions identical across study groups?	4. Were research personnel blinded to the study group during the study?	5. Were outcome data complete without attrition or exclusion from analysis?	6. Can we be confident in the exposure characterization?	7. Can we be confident in the outcome assessment (including blinding of assessors)?	8. Were all measured outcomes reported?	9. Were there no other potential threats to internal validity?
Bai et al. (2013) [12]	++ (no need for randomization)	++ (no need to be concealed)	++	++	++	++	++	++	++
Ottonello et al. (1996) [13]	++ (no need for randomization)	++ (no need to be concealed)	++	++	++	++	++	++	++
Komohara et al. (2013) [14]	++ (no need for randomization)	++ (no need to be concealed)	++	++	++	++	++	++	++
Miguet et al. (2006) [15]	+	+	++	++	++	++	++	++	++

Discussion

The complement system is not only a component of innate immunity and an ancient defense mechanism against invading pathogens but also an active participant in adaptive immune response, inflammation, hemostasis, and organ repair [4]. This involvement in many pathophysiological mechanisms makes the complement cascade an ideal target for approved and developing targeted therapies in several hematological entities, such as paroxysmal nocturnal hemoglobinuria, atypical hemolytic uremic syndrome, cold agglutinin disease, and transplant-associated thrombotic microangiopathy. However, the vast majority of these treatments are aimed at avoiding the formation of the membrane attack complex (MAC) on the surface of target cells and not chemotaxis caused by anaphylatoxins [16,17].

C3a and C5a Receptors

Anaphylatoxin receptors C3aR and C5aR are normally present on epithelial cells, mast cells, and phagocytes, and binding of C3a and C5a, respectively, enhances phagocytosis of opsonized microorganisms [18]. Many lymphoid malignant cell lines also express functional C3aR and C5aR receptors, which seem to respond to C3a and C5a by chemotaxis and increased adhesion [19]. Thus, the hypothesis that the above complement fragments may accelerate tumor growth through chronic inflammation seemed reasonable. Markiewski et al. proposed that C5a interacts with myeloid-derived suppressor cells (MDSCs), as they also express C5a receptors. Activated MDSCs migrate to tumors and produce highly immunosuppressive reactive oxygen species (ROS) and reactive nitrogen species (RNS) that inhibit the antitumor T-cell responses [20].

Inhibition of complement signalling has been studied thoroughly in malignant solid tumors, where pharmacological blockage of C5a receptors in tumor-bearing mice resulted in impaired tumor growth compared to controls. It was remarkable that the inhibition of complement signalling via the subcutaneous administration of C5aR antagonist in those mice significantly diminished tumor growth to a similar degree as the effect caused by the anti-cancer drug paclitaxel [20].

C5a-Mediated Proliferation of Lymphoma Cells

Additional studies in animals suggested that the concentration of local anaphylatoxins within the TME is critical in determining their role in tumor progression. It has been observed that tumor-bearing mice with high C5a-producing lymphoma cells had significantly accelerated tumor progression with decreased CD4+ and CD8+ T cells in the tumor, whereas mice with low C5a-producing lymphoma cells had a significantly reduced tumor burden with increased IFN-γ-producing CD4+ and CD8+ T cells in the spleen and tumor-draining lymph nodes [21]. The role of C5a in neutrophil chemotaxis and its significance in effective tumor regression by immunotherapy and β-glucan were also confirmed in lymphoma-bearing mouse models [22].

In another study, in lymphoma-bearing mice, the use of vaccines against an epitope of C5a receptor (C5aR) on antigen-presenting cells (APCs) showed impressive protective outcomes against an aggressive murine lymphoma. This connection between those vaccines and the C5aR epitope promoted the release of Th1 cytokines, which help drive the tumor-specific, CD8+ T-cell-mediated cytotoxic response [23].

In this systematic review, C5a appeared to promote the significant activation of lymphoma cell lines when co-cultured with M2 macrophages. More specifically, C5a was found to be one of the macrophage-derived soluble factors that induce cell-cell interaction between tumor-associated macrophages and lymphoma cells in ATLL and Burkitt lymphoma cell lines. Both Bai et al. [12] and Komohara et al. [14] suggested the existence of unknown signals other than C5a that also regulate this cell-cell interaction.

In Ottonello et al.'s in vitro study, a different mechanism is described where C5a, acting as a mediator, induced Lym-1 antibody-dependent cellular toxicity by polymorphonuclear leukocytes against lymphoma cells. In this study, Lym-1, a murine IgG2a monoclonal antibody that recognizes a polymorphic variant of HLA-DR antigens present on malignant B cells that has been given intravenously in previous clinical trials, achieved lymphoma cell lysis in the presence of C5a, whereas C5a-deficient human serum did not promote Lym-1 antibody-dependent lysis [13,24].

Diagnostic Biomarker Potential of C3a

Miguet et al. observed that C3a levels were increased in the serum of untreated lymphoma patients compared to the control group, suggesting the activation of the complement system via antigen-antibody complexes (classical pathway). In the same study, it was suggested that C3a and C4a might be potential biomarkers of B-cell lymphoma because they were identified in 38% of patients' serum with major peaks, but they were absent in the serum of the control group [15]. However, there was no further information concerning the role of C3a from the rest of the studies [12-14].

Limitations

Regarding the limitations of the study, despite an extensive search, a very limited number of studies were found to test this specific hypothesis. Evidence of a correlation between anaphylatoxin levels and lymphoma cell proliferation raises the need for further investigation in order to understand the role of anaphylatoxins in the turnover regulation of these cells. Ideally, studies that would correlate anaphylatoxin levels in patient serum with outcomes concerning survival and response to treatment would add valuable knowledge to this field.

## Conclusions

Anaphylatoxins, especially C5a, appear to play a role in the activation of lymphoma cells both as mediators in cell-cell interaction and as auxiliary molecules for the lysis of these cells via antibody-dependent cellular cytotoxicity. There are only few evidence about the effect of C3a on lymphoma. It is known that C3a is elevated in lymphoma patients' serum versus healthy subjects, but the reason for this increase has not been explained yet. The study of C3a interaction with lymphoma cells might prove useful in the search for more treatment options for lymphoma. Moreover, there seems to be a connection between M2 macrophages and C5a that promotes the proliferation of lymphoma cells. Also, downregulation of Stat3 stopped lymphoma cell activation by C5a. Exploring the pathophysiology between C5a, Stat3, and M2 macrophages may lead to a new breakthrough in the treatment of lymphoma. Further studies in this area could shed more light on the mechanisms by which anaphylatoxins affect lymphoma cells and maybe provide a new treatment strategy for lymphoma patients.
